# Association between gait speed and errors on the Clock Drawing Test in older adults with mild cognitive impairment

**DOI:** 10.1038/s41598-022-14084-2

**Published:** 2022-06-15

**Authors:** Hiroyuki Umegaki, Yusuke Suzuki, Hitoshi Komiya, Kazuhisa Watanabe, Masaaki Nagae, Yosuke Yamada, Masafumi Kuzuya

**Affiliations:** 1 Department of Community Healthcare and Geriatrics, Nagoya University Graduate School of Medicine, 65 Tsuruma-cho, Showa-ku, Nagoya, Aichi 466-8560 Japan; 2 Center for Community Liaison and Patient Consultations, Nagoya University Hospital, 65 Tsurumai-cho, Showa-ku, Nagoya, Aichi 466-8560 Japan

**Keywords:** Neurology, Signs and symptoms

## Abstract

Individuals with mild cognitive impairment (MCI) often make qualitative errors on the Clock Drawing Test (CDT), and these errors are reported to be associated with lower scores on neuropsychological assessments. Gait speed is also closely associated with cognitive dysfunction. However, the association between CDT errors and gait speed has not been investigated in individuals with MCI. Therefore, in this study, we explored the association between gait speed and qualitative errors on the CDT. Participants were 196 outpatients at a memory clinic with a clinical dementia rating of 0.5. The CDT was evaluated using the method of Cahn et al. The participants were divided into tertiles of normal and maximum gait speeds. The CDT error types of stimulus-bound response, conceptual deficit (CD), and planning deficit were found in 24.5%, 29.6%, and 30.1% of the participants, respectively. CD was found in 43.6% of the slowest tertile of maximum gait and in 22.2% of the fastest tertile. Multiple linear regression analysis gait speeds as objective continuous variables revealed that CD was significantly negatively associated with maximum gait, but not with normal gait. No other error types were associated with gait speeds. Only CD type error on the CDT was negatively associated with maximum gait speed, but not normal gait speed in the current study. The association between the qualitative error on the CDT and gait speed provides further basis of the clinical importance of qualitative assessments of CDT.

## Introduction

The Clock Drawing Test (CDT) has long been used as a screening test for dementia, and its validity has been confirmed. Several scoring systems for the CDT have been proposed^1^. Quantitative scoring uses a numerical scale to assess the drawing, while qualitative scoring evaluates characteristic errors in drawing the clock. Several error types are identified in the qualitative scoring systems^1^.

People with typical cognition also often make errors on the CDT. We previously reported that individuals with typical cognition and those with mild cognitive impairment (MCI) made CDT errors^2^, evaluated using Cahn et al.’s method^3^, and that these errors were associated with lower scores on neurocognitive assessments^2^. We have also reported an association between the conceptual deficit (CD) error type on the CDT in non-demented older adults and subsequent progression to dementia^4^.

We also previously reported that physical function and cognitive function were closely associated and declined in parallel^5^. Gait speed is a good marker of physical function and is easy to measure. Reduced gait speed is considered to be associated with cognitive impairment^6,7^ and cognitive decline^8^. Therefore, specific qualitative errors in the CDT, especially CD, may be associated with slow gait speed. However, to date, the association between gait and CDT error has not been explored. Gait speed is associated with executive dysfunction^6,9^. In a previous study, we found that subjects with MCI, as defined by Petersen’s criteria^10^, who had qualitative errors on the CDT had lower scores in multiple cognitive domains, including executive function^2^. Therefore, there may be an also association between qualitative errors on the CDT and slow gait speed. However, no studies have explored these associations although a study reported the trend of association between lower total scores of the CDT and slower gait speed^11^. It is thus warranted to determine the association between qualitative errors on the CDT and gait speed in order to understand the clinical features of MCI in individuals who make qualitative errors on the CDT. It may also provide further basis of the clinical importance of qualitative assessments of CDT to elucidate association between qualitative errors on the CDT and gait features.

In this study, we examined the association of the gait speeds with the types of CDT errors made in MCI individuals.

## Methods

### Participants

Among participants attending a memory clinic at a university hospital in Japan, those with a clinical dementia rating (CDR) score of 0.5^12^ were included. A CDR score of 0.5 has been used as a criterion for diagnosing MCI^13^. All participants were Japanese. The exclusion criteria were current serious medical or psychiatric disorder, history of symptomatic cerebrovascular disease, and gait disturbance due to articular disorders or needing an assistive device to walk. The study protocol was approved by the Ethics Committee of the Graduate School of Medicine, Nagoya University (2015-04-356977). Written informed consent was obtained from all participants. All methods were performed in accordance with the relevant guidelines and regulations. The registration of the subjects for the study is ongoing, and the current study included the subjects who were registered from January 2017 to December 2020. During this period the total of 261 subjects were diagnosed as CDR of 0.5 in the clinic, out of them 216 subjects participated the study. In the current analysis 23 subjects who lacked gait speeds data were excluded. The total of 193 subjects was included in the analysis.

### CDR

The CDR is widely used to determine the clinical stage of cognitive impairment^12,13^. Ratings are assigned based on information received from the subject and an accompanying informant on six subscales (memory, orientation, home and hobbies, judgement and problem solving, community affairs, and personal care). Each subscale is rated on a scale of 0–3 (0 = no impairment, 3 = need for maximal assistance), with a global CDR range of 0–3 (0 = a normal healthy individual with no cognitive or functional deficits; 0.5 = a normal healthy individual but with questionable cognitive and/or functional abilities indicating MCI; 1 = mild dementia; 2 = moderate dementia; and 3 = severe dementia). CDR was assessed by a well-trained and experienced nurse who was blinded to the CDT assessments. Participants in this study had global CDR scores of 0.5.

### Neuropsychological assessments

Three experienced clinical neuropsychologists administered the Mini Mental State Examination (MMSE)^14^ and the Alzheimer’s Disease Assessment Scale (ADAS) for general cognitive function^15^. Depressive mood was evaluated by Geriatric depression scale-15 (GDS-15)^16^ because depressive mood is reportedly a risk for slow gait^17^. The GDS-15 was divided into 2 groups at the cut-off point of 5/6 because of skewed distribution^18^

### Clock Drawing Test

The neuropsychologists administered the CDT. Participants were given a blank piece of paper and instructed to draw a clock face measuring 10 cm in diameter, write all the numbers on it, and then draw hands indicating a time of 10 past 11. The neuropsychologists then independently scored the drawn clocks using the method of Cahn et al.^3^, whereby both quantitative and qualitative scores are assessed independently. Quantitative scores were determined for three components—integrity of the clock face (0–2 points), absence and sequencing of the numbers (0–4 points), and presence and sequencing of the hands (0–4 points)—and qualitative scores were determined for the eight error types: (1) stimulus-bound response (SB); (2) conceptual deficit (CD), where the error reflects a loss or deficit in accessing knowledge about the attributes, features, and meaning of a clock; (3) perseveration (PR), where the activity continues or recurs without an appropriate stimulus; (4) neglect of left hemi-space (NL), where the clock’s attributes are written only on the right half of the clock face; (5) planning deficit (PD), where there are gaps before the numbers 12, 3, 6 or 9; (6) nonspecific spatial error (NS), where there is no specific pattern in the disorganization of the spatial layout of numbers; (7) numbers written outside of the clock (OC), where the numbers are written either on or around the perimeter; and (8) numbers written counterclockwise (CC), where the numbers are arranged with 12 at the top and then continuing counterclockwise^3^. Total CDT score was calculated by subtracting the sum of qualitative scores from the sum of quantitative scores, with a maximum score of 10. The higher the score, the better the performance on the CDT. Fleiss’s kappa of three raters for the sum of qualitative scores on CDT was 0.731, and, those for qualitative assessment for SB, CD, and PD were, 0.884, 0.866, and 0.850, respectively.

### Physical assessments

Normal and maximum gait speeds and the variables that can be associated with gait speed were assessed [grip strength, body mass index (BMI), and skeletal muscle mass index (SMI)].

#### Gait speed

Gait speed (m/s) was measured using a stopwatch. Participants were instructed to walk 5 m on a flat, straight path twice at their preferred speed and twice at maximum speed, with the average times calculated for each. Two markers were used to indicate the start and end of the walking path. Participants walked a 1-m section in advance of the start marker to ensure that they were walking at a comfortable pace by the time they reached it and the stopwatch was started.

#### Grip strength

Muscle strength which can be associated with gait was assessd. Grip strength in the participant’s dominant hand was measured using a portable grip strength dynamometer (GRIP-D; Takei, Niigata, Japan). The cut-off point for handgrip strength was < 28 kg for men and < 18 kg for women, based on the 2019 consensus update by the Asian Working Group for Sarcopenia^19^. Physical assessments were performed by nurses who were blinded to the neuropsychological assessments, including the CDT.

#### Body composition analysis

BMI was calculated by body weight and height. The amounts of extracellular and intracellular water in the body were directly measured using electrical current generated at three frequencies (5, 50, and 250 kHz) using a bioelectrical impedance data acquisition system (Inbody 430; Biospace Co., Seoul, South Korea). The system determines appendicular skeletal muscle mass from the segmental body composition and muscle mass. The SMI was calculated by dividing participants’ muscle mass by their height in meters squared (kg/m^2^). The cut-off for low SMI was < 7.0 kg/m^2^ for men and < 5.7 kg/m^2^ for women, based on the 2019 consensus update by the Asian Working Group for Sarcopenia^19^.

### Statistical analysis

The participants were divided into tertiles according to normal and maximum gait speeds. Background characteristics were shown according to tertiles of gait speeds. Because both of normal and maximum gait speeds distributed normally, multiple regression analysis using gait speeds as objective continuous variables were performed with adjustment of variables significantly associated in the univariate analysis to check for significant associations (*p* < 0.05). In the multiple regression for maximum gait speed, 4 statistical models with significant variables in the univariate analysis were conducted. model 1 was with adjustment of age and sex, and model 2 further included BMI, and model 3 included muscular mass (SMI) and strength (grip) instead of BMI, and finally model 4 included a cognitive variable into model 3.

## Results

The characteristics of the participants (n = 196) are shown in Table 1. The mean age was 78.6 ± 6.9 years, and 46.1% were men. The mean scores of MMSE and ADAS were 25.5 ± 2.7 and 10.0 ± 4.5, respectively. The mean normal and maximum gait speeds were 1.00 ± 0.23 and 1.45 ± 0.34 m/s, respectively.

**Table 1:** Participant characteristics.

Number	196
Age	78.6 ± 6.9
Male	46.10%
Years of education	12.5 ± 2.8
ADAS	10.0 ± 4.5
MMSE	25.5 ± 2.7
GDS-15	4.0 ± 2.9
Below cut-off of GDS-15	27.8%
BMI (kg/m^2^)	22.5 ± 3.3
SMI (kg/m^2^) male	7.3 ± 0.8
Female	5.7 ± 0.7
Below cut-off of SMI	40.20%
Maximum gait speed (m/s)	1.45 ± 0.34
Normal gait speed (m/s)	1.00 ± 0.23
Grip male	28.6 ± 6.7
Female	17.2 ± 4.8
Below cut-off of grip	51.0%
SB (%)	24.5
CD (%)	29.6
PD (%)	30.1

Mean ± Standard deviation was shown.

Cut-off point for GDS-15 was 5/6. Cut-off point for handgrip strength of < 28 kg for men and < 18 kg for women; cut-off for low SMI was < 7.0 kg/m^2^ in men and < 5.7 kg/m^2^ in women.

*ADAS* Alzheimer’s disease assessment scale, *MMSE* mini mental state examination, *GDS* geriatric depression scale, *BMI* body mass index, *SMI* skeletal muscle mass index, *SB* stimulus bound, *CD* conceptual deficits, *PD* planning deficits.

**Figure 1:**
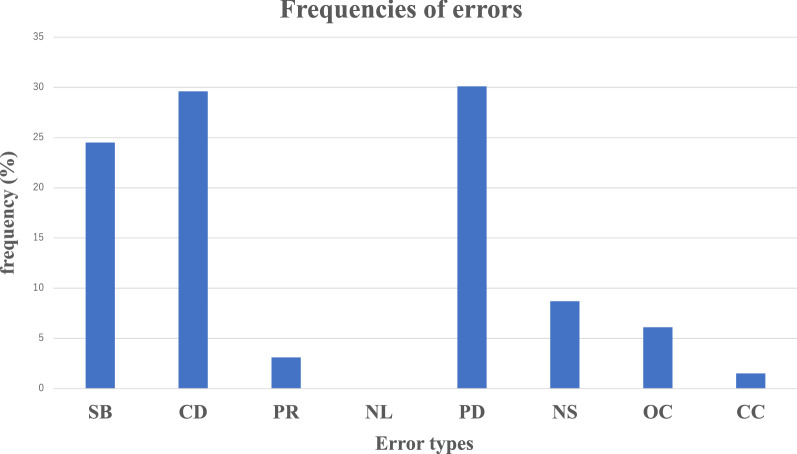
Frequencies of error types on the Clock Drawing Test. *SB* stimulus-bound response, *CD* conceptual deficit, *PR* perseveration, *NL* neglect of left hemi-space, *PD* planning deficit, *NS* nonspecific spatial error, *OC* numbers written on outside of the clock, *CC* numbers written counterclockwise.

The CDT error types SB, CD, and PD occurred most frequently (24.5%, 29.6%, and 30.1%, respectively) among the error types (PR, 3.1%; NS, 8.7%; OC, 6.1%; and CC, 1.5%; Fig. 1). None of the participants made NL errors.

The participants were divided into tertiles according to normal or maximum gait speed (Tables 2 and 3). The means or distributions of the variables were shown according to slowest, middle, and fastest tertiles of normal (Table 2) and maximum (Table 3) gait speeds. CD was found in 43.6% of the slowest tertile of maximum gait and in 22.2% of the fastest tertile (Table 2). SB and PD were found in about 20% and 30%, respectively, of all tertiles of maximum speed (Table 2). SB and CD were found in 27.8% and 35.2%, respectively of the slowest tertile of normal speed and in 18.8% and 26.6% of the fastest tertile (Table 3). PD was lower in the slowest tertile of normal gait (24.1%) compared with the fastest tertile (35.9%).

**Table 2:** Characteristics of participants in tertiles of maximum gait speed.

	Tertile 1 (slowest)	Tertile 2 (middle)	Tertile 3 (fastest)
Number	63	65	68
Age	81.5 ± 4.3	79.3 ± 5.1	74.1 ± 8.0
Sex (male)	33.30%	46.20%	57.40%
Years of education	12.2 ± 2.9	12.8 ± 2.8	12.9 ± 2.7
ADAS	10.8 ± 4.4	10.4 ± 4.3	8.5 ± 4.3
MMSE	24.9 ± 2.5	25.5 ± 2.7	26.5 ± 2.9
GDS-15	4.9 ± 3.3	3.7 ± 2.5	3.6 ± 2.8
Below cut-off of GDS-15	24.4%	28.4%	22.9%
BMI (kg/m^2^)	21.7 ± 3.2	22.3 ± 2.9	22.5 ± 3.3
SMI (kg/m^2^) male	7.1 ± 1.4	7.0 ± 0.7	7.5 ± 0.7
Female	5.9 ± 0.8	5.5 ± 0.7	5.7 ± 0.7
Below cut-off of SMI	52.7%	45%	25.8%
Maximum gait speed (m/s)	1.07 ± 0.14	1.41 ± 0.09	1.81 ± 0.22
Normal gait speed (m/s)	0.79 ± 0.14	0.99 ± 0.16	1.19 ± 0.18
Grip male	25.0 ± 4.3	24.0 ± 5.9	26.8 ± 6.0
Female	15.4 ± 3.9	17.5 ± 4.5	19.2 ± 4.8
Below cut-off of grip	68.3%	57.8%	26.5%
SB (%)	21.8	24.6	22.2
CD (%)	43.6	24.6	22.2
PD (%)	29.1	34.4	27

Mean ± Standard deviation was shown.

Cut-off point for GDS-15 was 5/6. Cut-off point for handgrip strength of < 28 kg for men and < 18 kg for women; cut-off for low SMI was < 7.0 kg/m^2^ in men and < 5.7 kg/m^2^ in women.

*ADAS* Alzheimer’s disease assessment scale, *MMSE* mini mental state examination, *GDS* geriatric depression scale, *BMI* body mass index, *SMI* skeletal muscle mass index, *SB* stimulus bound, *CD* conceptual deficits, *PD* planning deficits.

**Table 3:** Characteristics of participants in tertiles of normal gait speed.

	Tertile 1 (slowest)	Tertile 2	Tertile 3 (fastest)
Number	61	67	68
Age	81.2 ± 4.4	79.3 ± 5.4	74.4 ± 7.8
Sex	50.80%	37.30%	47.10%
Years of education	12.3 ± 3.0	12.8 ± 2.8	12.6 ± 2.7
ADAS	10.6 ± 4.6	10.8 ± 4.3	8.2 ± 3.9
MMSE	25.2 ± 2.5	25.0 ± 2.4	26.6 ± 2.8
GDS-15	4.5 ± 3.4	4.0 ± 2.5	3.6 ± 3.0
Below cut-off of GDS-15	28.3%	27.7%	18.6%
BMI (kg/m^2^)	22.2 ± 3.0	22.0 ± 3.2	23.3 ± 3.6
SMI (kg/m^2^) male	7.1 ± 1.4	7.1 ± 0.7	7.1 ± 0.8
Female	5.6 ± 0.7	5.5 ± 0.7	6.1 ± 0.7
Below cut-off of SMI	43.9	60.7	18.2
Maximum gait speed (m/s)	1.14 ± 0.23	1.41 ± 0.10	1.74 ± 0.28
Normal gait speed (m/s)	0.74 ± 0.11	0.98 ± 0.06	1.24 ± 0.13
Grip male	25.1 ± 5.5	28.8 ± 6.1	32.6 ± 6.6
Female	15.0 ± 4.0	16.9 ± 4.3	19.4 ± 4.8
Below cut-off of grip	73.3	44.8	34.8
SB (%)	27.8	22.2	18.8
CD (%)	35.2	27	26.6
PD (%)	24.1	30.2	35.9

Mean ± Standard deviation was shown.

Cut-off point for GDS-15 was 5/6. Cut-off point for handgrip strength of < 28 kg for men and < 18 kg for women; cut-off for low SMI was < 7.0 kg/m^2^ in men and < 5.7 kg/m^2^ in women.

*ADAS* Alzheimer’s disease assessment scale, *MMSE* mini mental state examination, *GDS* geriatric depression scale, *BMI* body mass index, *SMI* skeletal muscle mass index, *SB* stimulus bound, *CD* conceptual deficits, *PD* planning deficits.

The results of regression analysis to investigate the association with maximum gait speed as a continuous variable are shown in Table 4. The CD error type was significantly negatively associated with maximum gait speed in the univariate analysis, and several other variables were associated with maximum gait speed (Table 4). Multiple regression analysis was performed with adjustment of variables significantly associated in the univariate analysis (*p* < 0.05). Four models were formulated, and CD was included in all 4 models as a main variable of interest. In model 1 (adjusted for age, sex, and educational years), model 2 (model 1 + BMI), and model 3 (model 1 + SMI and grip), CD was significantly negatively associated with maximum gait (b = − 0.130, − 0.129, − 0.132 in models 1, 2, and 3, respectively). Model 4 (model 3 + ADAS) was just shy of statistical significance (b = − 0.132, *p* = 0.079). Including MMSE instead of ADAS did not change the trend. The vales of coefficients of variation (R^2^) for each model (models 1–4) were 0.353, 0.354, 0.405, and 0.416, respectively. Adjusted R^2^ for models 1–4 were 0.338, 0.335, 0.383, and 0.391, respectively. *p* Vales for F tests for all 4 models were < 0.001. No significant associations were found between CDT errors and normal gait speed (Table 5).

**Table 4:** Regression analysis for maximum gait speed.

	Univariate		Multivariate							
			Model 1		Model 2		Model 3		Model 4	
	*β*	*p* value	*β*	*p* value	*β*	*p* value	*β*	*p* value	*β*	*p* value
Age (year)	− 0.500	< 0.001	− 0.515	< 0.001	− 0.526	< 0.001	− 0.491	< 0.001	− 0.454	< 0.001
Sex (male = 1)	− 0.208	0.004	− 0.223	< 0.001	− 0.231	< 0.001	− 0.237	0.022	− 0.261	< 0.001
Years of education	0.179	0.013	0.059	0.365	0.058	0.373	0.039	0.551	0.022	0.737
BMI (kg/m^2^)	0.184	0.011			− 0.032	0.632				
SMI	− 0.236	0.001					0.065	0.346	0.077	0.262
Grip	− 0.343	< 0.001					− 0.198	0.003	− 0.194	0.003
ADAS	− 0.263	< 0.001							− 0.122	0.078
MMSE	0.337	< 0.001								
GDS-15	− 0.138	0.056								
SB	− 0.076	0.309								
CD	− 0.178	0.017	− 0.130	0.038	− 0.129	0.041	− 0.132	0.038	− 0.112	0.079
PD	− 0.038	0.615								

Cut-off point for GDS-15 was 5/6; Cut-off point for handgrip strength of < 28 kg for men and < 18 kg for women; cut-off for low SMI was < 7.0 kg/m^2^ in men and < 5.7 kg/m^2^ in women.

Model 1 (adjusted for age, sex, and educational years), model 2 (model 1 + BMI), model 3 (model 1 + SMI and grip), model 4 (model 3 + ADAS).

*ADAS* Alzheimer’s disease assessment scale, *MMSE* mini mental state examination, *GDS* geriatric depression scale, *BMI* body mass index, *SMI* skeletal muscle mass index, *CI* confidential interval, *SB* stimulus bound, *CD* conceptual deficits, *PD* planning deficits.

**Table 5:** Univariate regression analysis for normal gait speed.

	*β*	*p* value
Age	− 0.465	< 0.001
Sex	− 0.079	0.267
Years of education	0.106	0.136
ADAS-J cog	− 0.233	< 0.001
MMSE	0.226	0.001
GDS-15	− 0.147	0.038
BMI	0.121	0.089
SMI	− 0.217	0.003
Grip	− 0.320	< 0.001
SB	− 0.068	0.362
CD	− 0.128	0.086
PD	0.098	0.191

Cut-off point for GDS-15 was 5/6. Cut-off point for handgrip strength of < 28 kg for men and < 18 kg for women; cut-off for low SMI was < 7.0 kg/m^2^ in men and < 5.7 kg/m^2^ in women.

*ADAS* Alzheimer’s disease assessment scale, *MMSE* mini mental state examination, *GDS* geriatric depression scale, *BMI* body mass index, *SMI* skeletal muscle mass index, *CI* confidential interval, *SB* stimulus bound, *CD* conceptual deficits, *PD* planning deficits.

## Discussion

In this study we found that in participants with MCI (CDR 0.5), the CD type of error on the CDT was significantly negatively associated with maximum gait speed, whereas no significant associations were found in CDT errors with normal gait speed. Other qualitative errors on CDT were not associated with slow gait speed. The association between CD type error on CDT and slow maximum gait was independent from muscle markers (SMI and grip).

The CD error refers to loss of knowledge about the features and meaning of a clock. Impairment of semantic memory is considered to be associated with this type of error, and the impairment is thought to be caused by dysfunction of the lateral temporal lobe^20^. A study using single-photon emission computed tomography suggested that CD may be associated with lower blood perfusion in certain brain regions, including the posterior entorhinal cortex, posterior cingulate cortex, and parahippocampal cortex^21^. Our previous study suggested that making the CD error was associated with the risk of developing dementia^4^. In that study, CD was associated with impairment in several cognitive domains in both non-demented older adults and those with Alzheimer’s Disease. In a review paper, Amodeo et al. concluded that among the eight CDT error types, CD errors reflect cognitive decline over time most effectively^22^. Slow gait is an established risk factor for dementia^8^, and the present finding that participants who made CD errors had slower maximum gait speed could suggest a common underlying mechanism for the finding in our previous study that CD errors predicted cognitive decline. Our previous study also showed that making CD errors was associated with lower executive function scores in individuals with MCI^2^. Executive dysfunction is also reported to be associated with slow gait speed^23^. Slow gait and executive dysfunction are also associated with falls^24^, and CD errors on the CDT were found to be associated with falls in older adults^25^. The multiple regression analysis showed that CD was negatively associated with maximum gait even with the adjustment of muscle mass and power (model 3 in Table 4), but adjustment with general cognition attenuated the association between CD and maximum gait (Model 4 in Table 4). It may support that cognitive decline in those who made CD type error was associated with slow maximum gait. Another interpretation of the association between CD and slow maximum gait speed is also suggested: CD is an early sign of cognitive decline^2^, and slow gait, especially when exerting maximum effort, is a marker of subtle decline in physical function^26^. Therefore, the association between CD and slow maximum gait may be interpreted as a manifestation of parallel decline in both cognitive and physical function.

We found the association of CD type error and maximum gait speed. This is in agreement with a previous study in which maximum gait speed showed better sensitivity compared with normal gait speed to discriminate lower cognitive status in community-dwelling older adults^27^ and another study in which maximum gait speed, but not normal gait speed, predicted cognitive decline^28^. We also previously reported that maximum gait speed was associated with a wider range of neuropsychological assessments compared with normal gait speed^6^.

A recent systematic review reported that depressive mood was a risk for slow gait^17^. In the current study depressive mood was significantly negatively associated with normal gait in the univariate analysis (Table 5), but in maximum gait it just missed the statistical significance (Table 4).

In this study we used a CDR of 0.5 to define MCI, whereas in a previous study^4^ we used Petersen’s criteria^9^. Both sets of criteria have been widely used for the diagnosis of MCI, and the population defined by these sets of criteria is reported to overlap but is not exactly the same^29,30^. Therefore, the association between slower maximum gait speed and MCI seen in the participants who made CD errors needs to be interpreted with caution. Petersen’s criteria focus on memory impairment, whereas the CDR assesses a wide range of cognitive domains. Slow gait is reported to be associated with executive dysfunction^29^, which is not included in Petersen’s criteria. Further study is needed to determine whether individuals with MCI as defined by Petersen’s criteria have similar characteristics to the participants in the present study.

In conclusion, we found that making CD errors on the CDT was significantly associated with slow maximum gait speed in older adults with MCI.
